# Outbreak of Dengue Virus Type 2 — American Samoa, November 2016–October 2018

**DOI:** 10.15585/mmwr.mm6747a5

**Published:** 2018-11-30

**Authors:** Caitlin J. Cotter, A. John Tufa, Stephanie Johnson, Mary Matai’a, Rebecca Sciulli, Kyle R. Ryff, W. Thane Hancock, Christian Whelen, Tyler M. Sharp, Magele Scott Anesi

**Affiliations:** ^1^Epidemic Intelligence Service, CDC; ^2^Division of Vector-Borne Diseases, National Center for Emerging and Zoonotic Infectious Diseases, CDC; ^3^Pacific Island Health Officers’ Association, Honolulu, Hawaii; ^4^Applied Epidemiology Fellowship, Council of State and Territorial Epidemiologists; ^5^American Samoa Department of Public Health; ^6^Hawaii Department of Health State Laboratories Division; ^7^Division of State and Local Readiness, Office of Public Health Preparedness and Response, CDC.

The U.S. territory of American Samoa has experienced recent outbreaks of illnesses caused by viruses transmitted by *Aedes* species mosquitoes, including dengue, chikungunya, and Zika virus. In November 2016, a traveler from the Solomon Islands tested positive for infection with dengue virus type 2 (DENV-2). Additional dengue cases were identified in the subsequent weeks through passive and active surveillance. Suspected dengue cases were tested locally with a dengue rapid diagnostic test (RDT) for DENV nonstructural protein 1 (NS1). Specimens from RDT-positive cases and patients meeting the dengue case definition were tested by real-time reverse transcription–polymerase chain reaction (real-time RT-PCR) at Hawaii State Laboratories. During November 2016–October 2018, a total of 3,240 patients were tested for evidence of DENV infection (118 by RDT-NS1 alone, 1,089 by real-time RT-PCR alone, and 2,033 by both methods), 1,081 (33.4%) of whom tested positive for dengue (19.5 per 1,000 population). All 941 real-time RT-PCR-positive specimens were positive for DENV-2. The monthly number of laboratory-confirmed cases peaked at 120 during December 2017. Among laboratory-confirmed dengue cases, 380 (35.2%) patients were hospitalized; one patient, who was transferred to American Samoa for care late in his illness, died. The public health response to this outbreak included disposal of solid waste to remove mosquito breeding sites, indoor residual spraying of pesticides in schools, reinforcement of dengue patient management education, and public education on mosquito avoidance and seeking medical care for symptoms of dengue.

## Epidemiologic and Laboratory Surveillance

American Samoa consists of five Pacific Ocean islands. Among the 55,519 persons who resided in American Samoa in 2010, nearly all (95%) lived on the largest island, Tutuila, which has a land area of 76.8 square miles.* Electronic surveillance for dengue, chikungunya, and Zika virus disease has been in place since 2016. Electronic health records at Lyndon B. Johnson Tropical Medical Center and three of the five regional health centers were reviewed weekly by automated query to identify patients with febrile illnesses. The surveillance definition for suspected dengue included 1) the presence of two or more of the following: fever, rash, arthralgia, vomiting, nausea, myalgia, malaise, or headache; 2) the words “dengue,” “viral syndrome,” or “thrombocytopenia” in the electronic health record; or 3) an *International Classification of Disease, 10th Revision* (ICD-10) code for “unspecified viral illness,” “mosquitoborne illness,” or “arboviral fever.” Dengue with warning signs and severe dengue were defined according to World Health Organization 2009 case definitions (*1*).

Local diagnostic testing for patients with suspected dengue was performed using an RDT for DENV NS1 and anti-DENV immunoglobulin M (IgM), the sensitivity and specificity of which varies by DENV type and geographic location (*2*). Specimens from RDT-positive patients and from patients meeting the suspected dengue case definition were tested at Hawaii State Laboratories by real-time RT-PCR.^†^ Specimens testing positive for detection of DENV nucleic acid were further tested at Hawaii State Laboratories by multiplex DENV real-time RT-PCR (*3*). Laboratory-confirmed specimens included those positive by real-time RT-PCR or positive for NS1 by RDT. Because of the possibility of extended duration of antiflavivirus IgM antibody, potential crossreactivity of anti-Zika virus IgM antibody with DENV antigen, and lack of evaluation of test performance in American Samoa, 434 patients who tested positive only by RDT-IgM were excluded from further analysis. Estimated incidence was calculated using laboratory-confirmed dengue cases and population denominators from publically available sources.

On November 2, 2016, a fisherman from the Solomon Islands was evaluated in Sua County, American Samoa, with fever, arthralgia, rash, and shortness of breath. The real-time RT-PCR assay was negative for Zika virus and chikungunya virus, but positive for DENV. Additional testing identified DENV-2. Two days after being evaluated, the patient departed American Samoa. Soon after, additional suspected cases from neighboring counties were reported.

**FIGURE 1:**
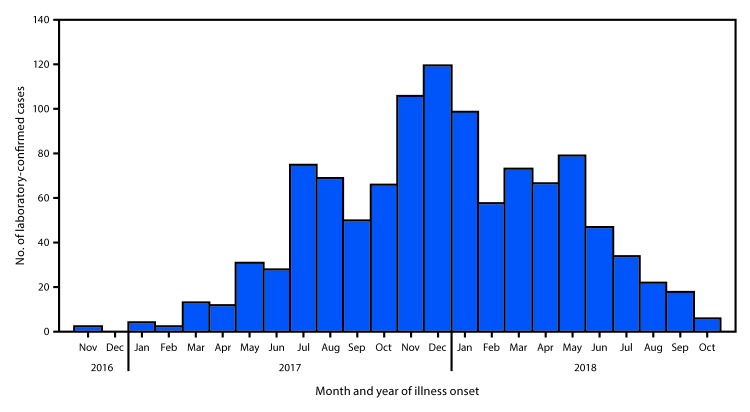
Laboratory-confirmed dengue cases (N = 1,079), by month of reported illness onset — American Samoa, November 2016–October 2018

After detection of the presumed index patient in November 2016, up to four laboratory-confirmed dengue cases were detected per month until March 2017, when case counts began to increase (Figure 1). The number of laboratory-confirmed cases detected per month reached 75 in July 2017, declined for 2 months, and increased again, peaking in December 2017 at 120 cases. The monthly number of laboratory-confirmed dengue cases gradually decreased from 96 in May 2018, and further decreased through October 2018, when six laboratory-confirmed cases were detected. The last identified laboratory-positive case reported illness onset on October 25, 2018.

Among 3,122 serum specimens tested by real-time RT-PCR, 941 (30.1%) were positive for DENV-2. No cases tested positive by real-time RT-PCR for Zika virus, chikungunya virus, or another DENV type. Among 2,151 specimens tested by RDT, 421 (19.6%) were positive for detection of NS1. A total of 281 cases tested positive by both real-time RT-PCR and RDT-NS1. Overall, 1,081 (33.4%) laboratory-confirmed dengue cases (19.5 per 1,000 population) were identified.

**FIGURE 2:**
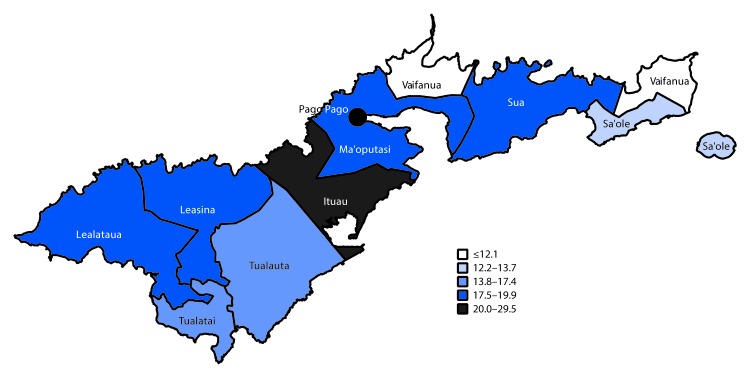
Number of laboratory-confirmed dengue cases per 1,000 persons, by county of residence — American Samoa, November 2016–October 2018

As of October 31, 2018, the incidence of laboratory-confirmed dengue cases by county was highest in Ituau County (29.5 per 1,000 population), which neighbors the county containing the capital city of Pago Pago (Figure 2). The incidence of laboratory-confirmed dengue cases among other counties ranged from 12.1 to 19.9 per 1,000 population. Among laboratory-confirmed cases, 50.6% of patients were female, and median age was 16 years (Table). Incidence of laboratory-confirmed dengue was highest among persons aged 10–19 years (38.1 per 1,000 population) and lowest among persons aged 40–49 years (10.6). Overall, 380 (35.2%) patients with laboratory-confirmed dengue were hospitalized. A man aged 68 years who had been transferred for care from neighboring Samoa died within 24 hours of arrival in American Samoa. Among 89 hospitalized laboratory-confirmed dengue patients for whom medical records were reviewed, 30 (33.7%) had dengue with warning signs, and 23 (25.8%) had severe dengue.

**TABLE:** Number and percentage of dengue patients (N = 1,081*), by selected characteristics and rate of cases per 1,000 population by age group — American Samoa, November 2016–October 2018

Characteristic	No. (%)
Female	547 (50.6)
Age, median (range)	16 yrs (0–87 yrs)
Travel outside of American Samoa within 14 days of illness onset	8 (0.7)
**Signs/Symptoms**	
Fever	994 (92.0)
Myalgia	687 (63.6)
Headache	525 (48.6)
Nausea	314 (31.5)
Vomiting	308 (28.5)
**Severity of disease among 89 hospitalized patients**	
Dengue with warning signs^†^	30 (33.7)
Severe dengue^§^	23 (25.8)
Fatal	0 (0.0)
**Age group (yrs)**	**Cases per 1,000 population** ^¶^
0–9	16.7
10–19	38.1
20–29	17.7
30–39	11.1
40–49	10.6
50–59	10.8
60–69	12.7
≥70	13.0

* Demographic data were missing for three cases.

^†^ Abdominal pain or tenderness, persistent vomiting, clinical fluid accumulation, mucosal bleed, lethargy, restlessness, liver enlargement >2 cm, increase in hematocrit concurrent with rapid decrease in platelet count.

^§^ One or more of the following: 1) plasma leakage leading to shock or fluid accumulation, with or without respiratory distress, 2) severe bleeding, or 3) severe organ impairment.

^¶^ Incidences calculated using laboratory-confirmed dengue cases and population denominators from the U.S. Census Bureau, 2011 American FactFinder. https://factfinder2.census.gov/faces/nav/jsf/pages/index.xhtml.

## Public Health Response

When American Samoa declared the DENV-2 outbreak in March 2017, the Zika public health response was ongoing, and those response efforts remained in effect and were applied to combat dengue. The Environmental Health Division of the American Samoa Department of Public Health (ASDOH) conducted detailed outdoor environmental assessments of private properties and business locations, issuing citations to those in violation of mosquito breeding site removal laws. During August–September 2017, an estimated 108 tons of solid waste and scrap metal were removed from yards and public spaces. The Environmental Health Division conducted indoor residual spraying in all public and private schools, focusing environmental inspections on private properties and businesses in the villages with the highest incidence of laboratory-confirmed cases. The American Samoa territorial epidemiologist spoke on broadcast radio and television programs to spread public messaging regarding seeking care for acute febrile illness and ways to prevent mosquito bites; educational messages were posted on billboards in high-traffic locations. Mosquito repellent sprays were distributed at community health clinics across the island.

## Discussion

Dengue is the world’s most common mosquitoborne viral disease (*1*), resulting in an estimated 58 million symptomatic infections and 13,000 deaths in 2013 (*4*). Approximately 75% of dengue virus infections do not result in illness (*5*); however, 5% of patients progress to severe dengue. The case-fatality rate among hospitalized dengue patients ranges from 0.5% to 5.0% (*1*), and the rate can be reduced by improving the timing and quality of clinical care (*6*).

During an outbreak of DENV-3 in American Samoa in 2015, approximately 900 suspected dengue cases were reported, including four laboratory-confirmed fatal cases (unpublished data, ASDOH). Clinical dengue management trainings were conducted by American Samoa and CDC in response to these fatalities, and no further DENV-3 associated deaths among persons infected in Samoa were reported. Through continued adherence to proper dengue management techniques, no fatal cases resulting from the current DENV-2 outbreak in American Samoa have been reported. Similar training can be considered for dengue outbreak responses in other locations.^§^

American Samoa has had numerous outbreaks of arboviral disease, starting with DENV-2 in 1972 (*7*). A serosurvey conducted in 2010 demonstrated 96% seroprevalence against DENV, suggesting widespread exposure among all age groups (*8*). Chikungunya virus was detected in American Samoa in 2014, followed by the outbreak of DENV-3 in 2015. Zika virus was first detected in American Samoa in January 2016 (*9*), and transmission was waning but still ongoing when the DENV-2 outbreak was detected in November 2016 (*10*).

Since the DENV-3 outbreak in 2015, ASDOH has used electronic arboviral disease surveillance on the island of Tutuila, which helped identify the apparent index patient in the most recent outbreak and stimulated a public health response. Because of limitations in patient care-seeking behavior, physician awareness, diagnostic sensitivity, and interpretation of RDT-IgM, the 1,081 laboratory-confirmed dengue cases likely underestimate the actual magnitude of this outbreak.

Transmission of DENV-2 continued in American Samoa for at least 24 months, demonstrating the need for sustainable and effective vector control interventions. Further efforts to develop and implement sustainable and effective vector control interventions are needed. Appropriate medical management appears to be effective at decreasing the number of dengue-related deaths. Persons living or traveling in areas with endemic dengue who develop an acute febrile illness should immediately seek medical care, and clinicians should be aware of appropriate testing and management for patients suspected of dengue (*1*) and other arboviral diseases. Persons residing or traveling in regions with endemic dengue should use insect repellent, wear long sleeves and pants, and stay in residences with screens on doors and windows where possible.^¶^

**Summary**

**What is already known about this topic?**

American Samoa has experienced multiple outbreaks of mosquitoborne viral disease in recent years, including chikungunya in 2014, dengue in 2015, and Zika in 2016.

**What is added by this report?**

During November 2016–October 2018, 1,081 laboratory-confirmed dengue cases were identified, with only dengue virus type 2 detected. The epidemic peaked in December 2017, after which, case counts slowly decreased.

**What are the implications for public health practice?**

Sustainable, effective interventions are still needed to control dengue, as is continued emphasis on clinical management to reduce mortality. Persons residing in or traveling to areas with risk for dengue should use insect repellent, wear long sleeves and pants, and stay in residences with screens on doors and windows.

## Footnotes

* http://factfinder2.census.gov/faces/nav/jsf/pages/index.xhtml.

† https://www.nature.com/articles/s41467-018-03772-1.

§
https://www.cdc.gov/dengue/educationtraining/index.html .

¶ https://www.cdc.gov/dengue/prevention/index.html.
